# F13. TOWARDS MOLECULAR INSIGHTS INTO PSYCHIATRIC DISORDERS USING AFFINITY PROTEOMICS

**DOI:** 10.1093/schbul/sby017.544

**Published:** 2018-04-01

**Authors:** David Just, Anna Månberg, Eva Lindholm Carlström, Janet Cunningham, Peter Nilsson

**Affiliations:** 1 Affinity Proteomics-Science for Life Laboratory, KTH-Royal Insititute of Technology; 2 Uppsala University

## Abstract

**Background:**

Numerous studies have shown a correlation between high autoantibody titers and subsequent autoimmune disease in patients with psychiatric disorders compared to healthy individuals. In this study we used a targeted affinity proteomics approach to investigate these autoantibody repertoires. We therefore obtained serum samples from patients diagnosed with various psychiatric disorders and compared these with samples of healthy volunteers. Additionally we used our approach to identify autoantibodies in post mortem brain tissue from patients diagnosed with schizophrenia.

**Methods:**

In this study we utilized a well characterized cohort of patients with psychiatric disorder to study the autoantibody repertoire. From this sample set we analysed more than 600 serum in a first discovery approach. Based on the in-house screening and previous external published studies of autoantibodies within psychiatry we selected a set of 231 protein fragments from the Human Protein Atlas with a length of roughly 80 amino acids. With this selected panel we screened additional 130 post mortem brain tissue samples. Autoantibody profiling was performed using suspension bead array technology and IgG reactivity was measured in patients and controls.

**Results:**

Our findings could indicate altered immune response in patients with psychiatric disorders compared to healthy controls. In our study we identified potential predictive autoimmune signatures. Those were presented with higher IgG reactivity in patients compared to healthy control samples.

**Discussion:**

With our approach we were able to profile autoantibody repertoires in patients with psychiatric disorders. Additionally, we were able to use our approach to profile brain tissue using a multiplexed affinity proteomics approach. By further validating these putative autoimmunity targets, we could gain insights into the autoantigens associated to chronical mental illnesses.

